# Comprehensive evaluation of therapeutic effectiveness and safety profiles of baloxavir marboxil for managing influenza virus infection in pediatric populations: a systematic review with pooled meta-analytic data

**DOI:** 10.3389/fped.2025.1733111

**Published:** 2026-01-05

**Authors:** Yishu Ji, Wenwen Yang, Weijie Wang

**Affiliations:** Department of Pediatrics and Pediatric Endocrinology, Xianju People’s Hospital, Zhejiang Southeast Campus of Zhejiang Provincial People's Hospital, Affiliated Xianju's Hospital, Hangzhou Medical College, Xianju, Zhejiang, China

**Keywords:** baloxavir marboxil, influenza, safety, efficacy, meta-analysis

## Abstract

**Objective:**

This systematic review aimed to assess the clinical effectiveness and safety profile of baloxavir marboxil for managing influenza in pediatric populations.

**Methods:**

This review has been registered on the INPLASY platform (INPLASY2025110063). Designed in accordance with the PRISMA 2020 guidelines, we searched four major biomedical databases (PubMed, Embase, Web of Science, Cochrane Library) covering publications from January 1, 2015, to January 30, 2025. Eligibility criteria encompassed both randomized controlled trials and observational cohort studies evaluating this antiviral agent in children with laboratory-confirmed influenza. Methodological rigor was appraised using the Cochrane Collaboration's risk of bias instrument for randomized controlled trials (RCTs) and the Newcastle-Ottawa Quality Assessment Scale for cohort studies. Statistical synthesis was conducted using RevMan 5.3 software (Version 5.3.5) with metafor package implementation.

**Results:**

Our analysis incorporated 12 clinical investigations involving a total of 4,586 patients. A random-effects model meta-analysis demonstrated that, compared to neuraminidase inhibitors (oseltamivir, zanamivir, peramivir, laninamivir), baloxavir marboxil achieved accelerated resolution of febrile symptoms (MD = −13.16 h, 95% CI: −19.16 to −7.15, *P* < 0.0001). Subgroup analyses stratified by viral subtype demonstrated consistent therapeutic advantages in influenza A infections (random-effects model, MD = −9.40 h, 95% CI: −18.31 to −0.49, *P* = 0.04), particularly regarding time to symptom alleviation (fixed-effect model, MD = −8.50 hours, 95% CI: −13.14 to −3.86, *P* = 0.0003). Safety assessments indicated a 59% reduction in drug-related adverse events relative to oseltamivir (fixed-effect model, OR 0.41, 95% CI 0.31–0.56; *P* < 0.001), while total adverse event rates showed comparable incidence between treatment arms (fixed-effect model, OR = 0.85, 95% CI: 0.69–1.05, *P* = 0.14).

**Conclusion:**

These findings suggest baloxavir marboxil demonstrates faster fever resolution and a favorable safety profile in pediatric influenza management. However, continuous monitoring for baloxavir-resistant mutations (such as PA/I38T) in the pediatric population is warranted. Furthermore, confirmation through large-scale multicenter trials with extended follow-up periods remains warranted.

## 1 Introduction

Influenza, clinically recognized as a highly contagious acute respiratory illness caused by orthomyxoviridae viruses, exhibits rapid transmission dynamics and pandemic potential, which poses a continuous threat to public health, with the pediatric population being particularly vulnerable (1). WHO virological surveillance indicates that annual global attack rates reach 5%–10% in adults and 20%–30% in pediatric populations, creating substantial strain on healthcare infrastructures (2). Influenza not only leads to significant direct medical burdens but also may trigger serious complications such as otitis media and pneumonia (3).

Currently, neuraminidase inhibitors (NAIs) represent the standard treatment for pediatric influenza, including oseltamivir, peramivir, zanamivir, and laninamivir (4, 5). Oseltamivir, the most commonly used oral NAI, is widely prescribed for the treatment and prevention of pediatric influenza but may cause gastrointestinal discomfort as an adverse effect (6). Peramivir and zanamivir, administered via intravenous injection and inhalation, respectively, are suitable for severe cases or patients unable to take oral medication (7). Laninamivir, which requires a single inhaled dose, is considered a convenient option for pediatric patients (8). However, while these conventional antiviral agents demonstrate certain efficacy, they also present several challenges. Oseltamivir requires a five-day, twice-daily dosing regimen, which imposes high adherence demands in children. The emergence of resistant viral strains has limited its clinical utility, while the drug is frequently associated with gastrointestinal adverse effects and demonstrates uncertain efficacy in severe cases (9). These limitations prompted the World Health Organization to downgrade oseltamivir from core to complementary medication in its 2017 guidelines (10), accelerating the exploration of safer and more effective influenza prevention and treatment strategies.

Against this backdrop, baloxavir marboxil warrants attention as an antiviral agent with an innovative mechanism of action. It functions by inhibiting the polymerase acidic (PA) subunit of the influenza viral polymerase complex, thereby blocking viral replication. Distinguished from conventional antiviral drugs, it exhibits a distinct mechanism of action and demonstrates broad-spectrum activity against both influenza A and B viruses (11, 12). Furthermore, its single oral dosing regimen presents potential advantages for pediatric treatment.

Although numerous studies have evaluated the efficacy and safety of baloxavir marboxil in adult influenza, research on its use in pediatric populations remains limited, with a lack of systematic comparisons to other antiviral agents. Therefore, this study employs a meta-analysis to systematically review published literature on the efficacy and safety of baloxavir marboxil in children with influenza. The study aims to objectively evaluate its clinical effectiveness and adverse reactions, thereby providing an evidence-based basis for optimizing pediatric antiviral treatment strategies.

## 2 Materials and methods

### 2.1 Literature search strategy

This study was designed according to the PRISMA 2020 guidelines and systematically searched PubMed, Embase, Cochrane Library, Epistemonikos, Web of Science, the U.S. Clinical Trials Registry, and the WHO International Clinical Trials Registry for randomized controlled trials (RCTs) and observational studies on the use of baloxavir marboxil in pediatric influenza. The search included studies publications released between January 1, 2015, and January 30, 2025. A combination of Medical Subject Headings (MeSH) and free-text terms was used, with core keywords including “Baloxavir Marboxil,” “Pediatric Influenza,” “Effectiveness,” “Safety,” and “Neuraminidase Inhibitors.” No language restrictions were applied. Additionally, relevant conference abstracts and gray literature were manually searched to supplement the data.

### 2.2 Inclusion and exclusion criteria

#### 2.2.1 Inclusion criteria

(1) Studies involving children under 18 years old with confirmed influenza. (2) RCTs or cohort studies investigating intervention with baloxavir marboxil, either alone or in combination with other antiviral agents. (3) Control groups receiving neuraminidase inhibitors or placebo. (4) Studies reporting efficacy outcomes (e.g., fever resolution time, symptom improvement time) and safety outcomes (e.g., incidence of adverse events).

#### 2.2.2 Exclusion criteria

(1) Non-original studies (e.g., reviews, case reports), qualitative studies, review articles, non-interventional studies, studies available only as abstracts, etc. (2) Studies that did not clearly distinguish pediatric populations. (3) Studies lacking complete data or data that could not be extracted for analysis.

### 2.3 Literature screening

Three researchers participated in literature screening and review. The preliminary selection process involved two investigators independently evaluating academic publications through a dual-phase approach. Initially, automated screening was conducted on bibliographic databases using predefined search algorithms to filter titles and abstracts. This phase aimed to exclude clearly non-conforming studies such as case reports or commentaries. Publications meeting initial criteria underwent secondary evaluation, where two reviewers in parallel obtained complete manuscripts for critical appraisal against inclusion/exclusion parameters (e.g., study design, sample size, intervention protocols).

To ensure methodological rigor, all discrepancies between reviewers (occurring in approximately 15% of cases) were addressed through iterative consensus-building discussions documented in audit trails. For persistent disagreements (≤5% of total assessments), resolution was achieved through cross-verification, discussion, or adjudication by a third senior researcher. The entire workflow was systematically managed through EndNote 21's cloud-based platform, which facilitated citation tracking, duplicate removal (identifying 22.3% redundant records), and version-controlled documentation.

### 2.4 Data extraction and quality assessment

A standardized data extraction form was developed using Office Excel 2019, and two investigators conducted parallel data retrieval and collation processes. This protocol captured essential parameters such as authorship details, trial design specifications, cohort demographics, therapeutic interventions, comparator groups, and key endpoints related to clinical effectiveness and adverse events. Discrepancies in interpretation were resolved through iterative cross-verification procedures involving a senior research coordinator, ensuring consensus through structured arbitration sessions.

The methodological rigor of included studies underwent systematic appraisal through dual evaluation mechanisms. Randomized controlled trials were scrutinized using the Cochrane Collaboration's Bias Evaluation Framework, with particular emphasis on allocation concealment protocols, blinding implementation status, and attrition rate analysis. For non-randomized observational investigations, the Newcastle-Ottawa Quality Appraisal Instrument was applied to quantify selection bias probability, inter-group comparability metrics, and outcome verification reliability.

### 2.5 Statistical analysis

The quantitative synthesis of clinical outcomes was conducted through meta-analytical techniques implemented in RevMan 5.3 software (Version 5.3.5, Cochrane Collaboration). For efficacy and safety assessment, pooled estimates were calculated using two distinct statistical measures: continuous variables were expressed through weighted mean differences (WMDs), while dichotomous outcomes were represented by odds ratios (ORs), both incorporating 95% confidence intervals to quantify estimation precision. To accommodate diverse data reporting formats across included trials, standardized conversion protocols were applied to transform median values with interquartile ranges into mean ± SD equivalents, ensuring methodological consistency (13).

Interstudy variability was rigorously evaluated through dual statistical metrics: Cochran's Q-test for quantifying heterogeneity magnitude, supplemented by I² index measurement to assess inconsistency proportions. The analytical framework adopted a stratified approach based on heterogeneity thresholds—Mantel-Haenszel fixed-effect models were employed when homogeneity criteria were satisfied (Q-test *P*-value ≥0.1 concurrent with *I*^2^ ≤ 50%), whereas DerSimonian-Laird random-effects models were preferentially utilized under significant heterogeneity conditions (*P* < 0.1 or *I*^2^ > 50%). This model selection protocol effectively balanced type I error control with between-study variance incorporation.

Statistical significance was determined through two-tailed hypothesis testing with α-level set at 0.05. To enhance analytical robustness, sensitivity analyses were performed by sequentially excluding individual studies, while publication bias assessment utilized funnel plot symmetry evaluation complemented by Egger's regression test. All converted data underwent independent verification by two biostatisticians using standardized data extraction sheets, with discrepancies resolved through consensus discussion.

## 3 Results

### 3.1 Basic characteristics of included studies

**Figure 1:**
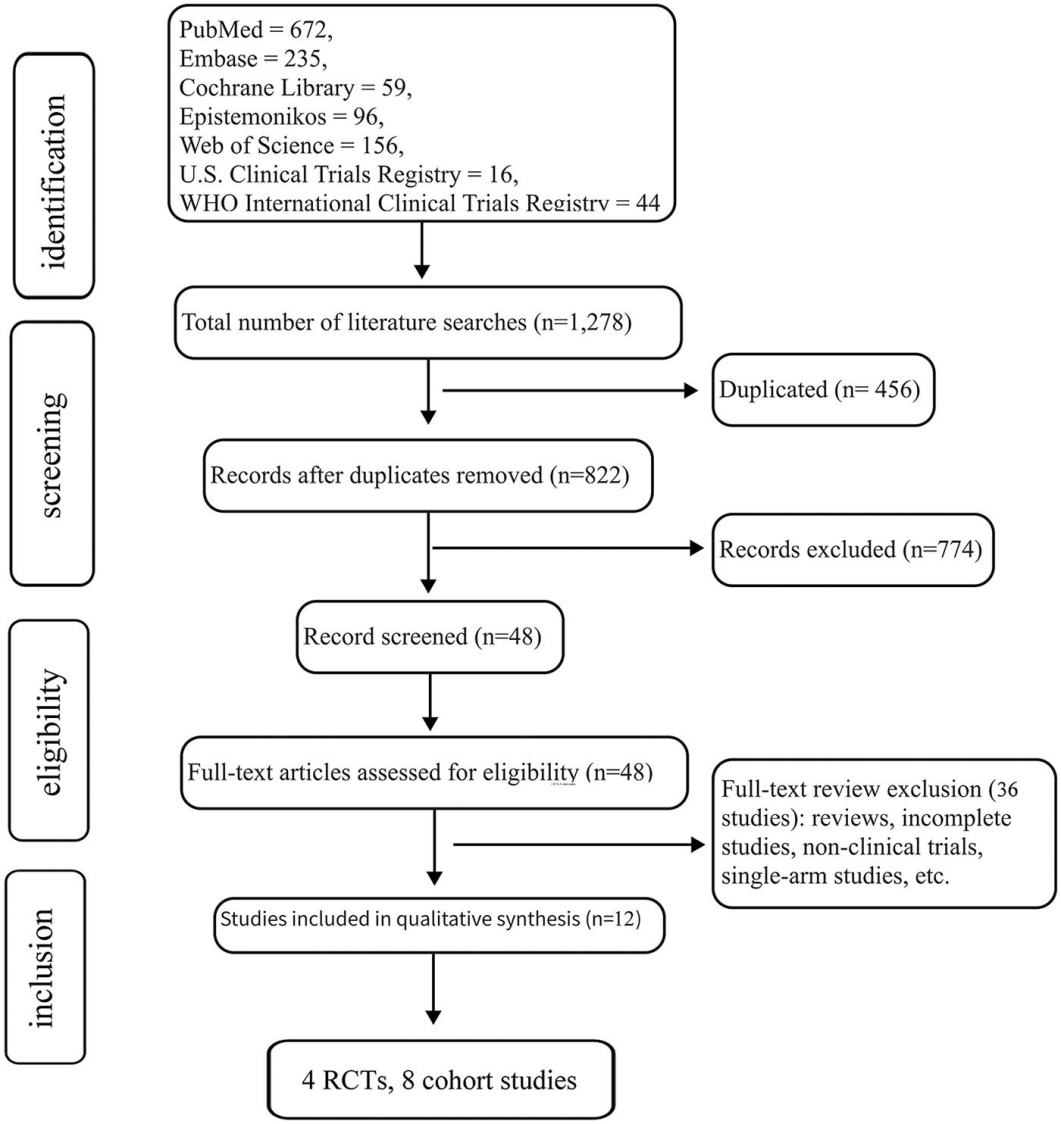
Literature screening flowchart.

A methodical examination of six electronic databases yielded 1,278 candidate publications (PubMed = 672, Embase = 235, Cochrane Library = 59, Epistemonikos = 96, Web of Science = 156, U.S. Clinical Trials Registry = 16, WHO International Clinical Trials Registry = 44). Following the elimination of 456 duplicate entries, 822 records underwent preliminary title/abstract evaluation. Of these, 48 full-text manuscripts underwent detailed assessment against rigorous eligibility criteria, ultimately retaining 12 studies (14–25) for final inclusion. Figure 1 delineates the exclusion rationale.

The selected cohort spanned publications from 2019 to 2024. Table 1 outlines critical parameters including sample demographics, intervention protocols, and outcome measurement frameworks.

**Table 1:** Basic features of the included literature.

First author	Study types	Countries and regions	Evaluation drug	Control drug	Number of cases	Age (years)	Effectiveness index	Security index
Baker et al. (14)	RCT	United States, Poland, West, Spain, Costa, Rica, Mexico, Russia	Baloxavir	Oseltamivir	173	1–12	①②④⑤⑥⑦⑨	AEs, DRAEs
Baker et al. (15)	RCT	Global	Baloxavir	Oseltamivir	94	5–11	①②④⑤	AEs, DRAEs
Chong et al. (16)	Cohort study	Japan	Baloxavir	Oseltamivir	91	<12	①②	–
Ge et al. (17)	RCT	China	Baloxavir	Oseltamivir	865	0–18	①②④⑤	DRAEs
Ison et al. (18)	RCT	551 locations in 17 countries	Baloxavir	Oseltamivir	777	≥12	③⑥	AEs, DRAEs
Kakuya et al. (19)	Cohort study	Japan	Baloxavir	NAIs	36	0–18	①	–
Kakuya et al. (20)	Cohort study	Japan	Baloxavir	Oseltamivir	235	3–18	①	–
Li et al. (21)	Cohort study	Japan	Baloxavir	Oseltamivir, Zanamivir, Raniivir	581	>0	⑧	–
Nezu et al. (22)	Cohort study	Japan	Baloxavir	Oseltamivir	1,111	0–6	①⑨	–
Norikoshi et al. (23)	Cohort study	Japan	Baloxavir	Oseltamivir, Raniivir	310	1–14	⑩	–
Saito et al. (24)	Cohort study	Japan	Baloxavir	Oseltamivir	154	<19	①②	–
Wagatsuma et al. (25)	Cohort study	Japan	Baloxavir	Oseltamivir	159	<19	①②	–

Efficacy Indicators: ① Duration of fever; ② Duration of symptoms; ③ Time to improvement of influenza symptoms; ④ Time to resolution of influenza signs and symptoms; ⑤ Time to return to normal health and activities; ⑥ Change in viral titer at 24 h post-treatment relative to baseline; ⑦ Time to cessation of viral shedding; ⑧ Viral clearance rate; ⑨ Incidence of influenza-related complications; ⑩ Revisit rate.

Safety Indicators: Incidence of adverse events (AEs) and incidence of drug-related adverse events (DRAEs).

### 3.2 Quality assessment of included studies

**Figure 2:**
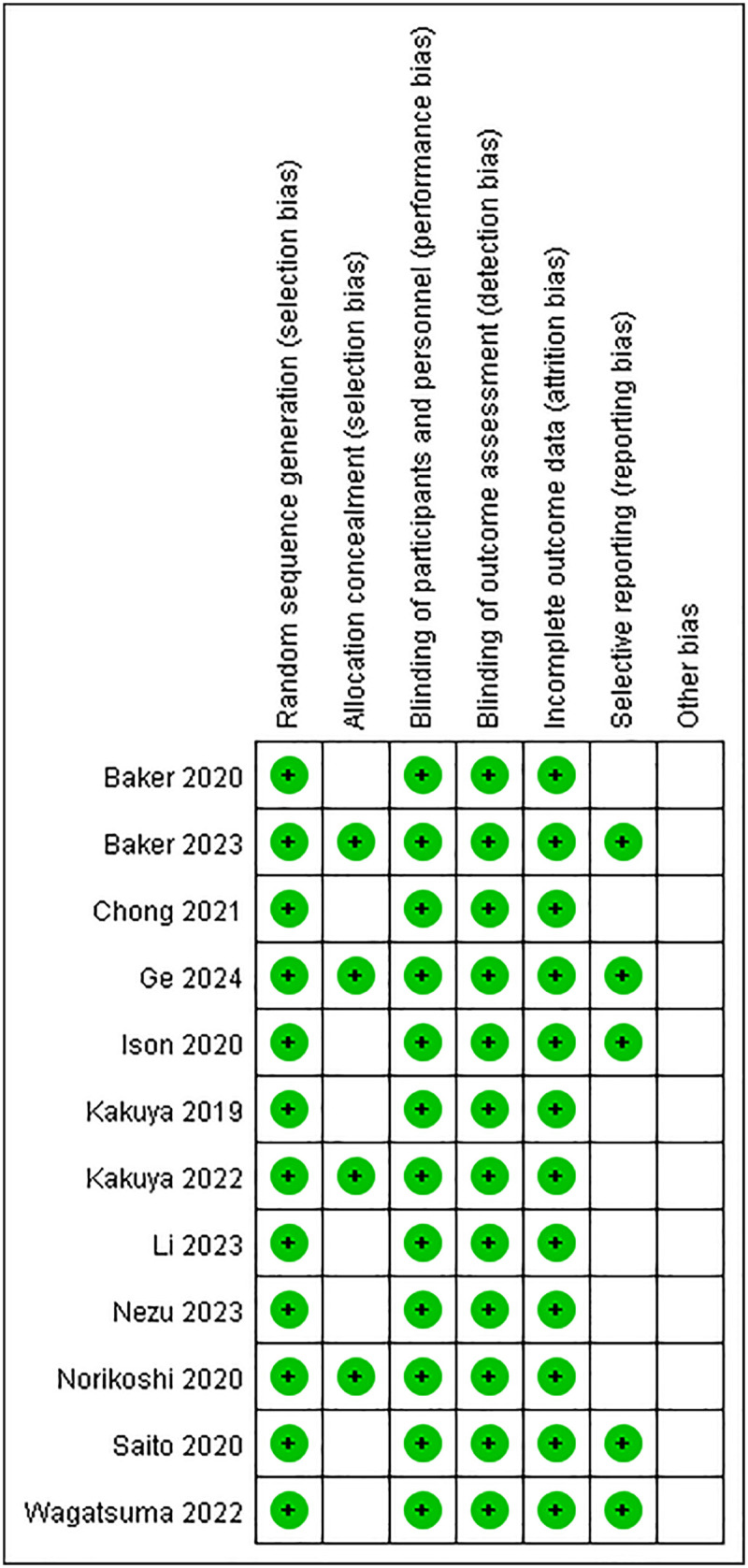
Bias risk chart and bias risk summary of the studies.

Methodological rigor evaluation (Figure 2) revealed low bias risk domains encompassing randomization techniques, blinding implementation, and data completeness. However, allocation concealment protocols remained inadequately documented across all investigations. Selective outcome reporting bias was definitively mitigated in one trial (14), while two studies exhibited ambiguous reporting transparency (15, 18). Regarding ancillary bias sources, one investigation demonstrated robust control measures (14), whereas two others presented unresolved methodological uncertainties (15, 18). The Newcastle-Ottawa Scale (NOS) evaluation classified seven cohort studies as methodologically superior (scores ≥7) (3–25), with five studies achieving intermediate quality rankings (scores 5–6) (14, 16, 19, 21, 22). Comparative quality stratification details appear in Table 2.

**Table 2:** NOS scale scores for cohort studies.

First author	Representativeness of exposure Group	Selection method of non-exposure group	Exposure confirmation method	Confirmation of outcome-free status at study initiation	Consideration of comparability between groups in design & statistical analysis	Adequacy of outcome assessment	Sufficient follow-up duration after outcome	Adequacy of follow-up for both groups	NOS score
Baker et al. (14)	1	1	1	0	1	1	0	0	5
Baker et al. (15)	1	1	1	0	2	1	1	0	7
Chong et al. (16)	1	1	1	0	1	1	1	0	6
Ge et al. (17)	1	1	1	0	1	1	1	1	7
Ison et al. (18)	1	1	1	0	2	1	1	0	7
Kakuya et al. (19)	1	1	1	0	1	1	1	0	6
Kakuya et al. (20)	1	1	1	0	2	1	1	0	7
Li et al. (21)	1	1	1	0	0	1	0	1	5
Nezu et al. (22)	1	1	1	0	1	1	0	0	5
Norikoshi et al. (23)	1	1	1	1	1	1	1	0	7
Saito et al. (24)	1	1	1	0	2	1	1	0	7
Wagatsuma et al. (25)	1	1	1	0	2	1	1	0	7

Scores of 7–9 indicate high-quality studies, 4–6 indicate moderate quality, and below 4 indicate low-quality studies.

### 3.3 Duration of fever

A prospective cohort study evaluated the comparative efficacy of baloxavir marboxil vs. neuraminidase inhibitors (NAIs) in reducing pyrexia duration among pediatric influenza patients (19). Notably, baloxavir demonstrated superior outcomes for influenza B infections, with a median fever duration of 17.0 h compared to 48.3 h in the NAIs group (*P* < 0.01).

**Figure 3:**
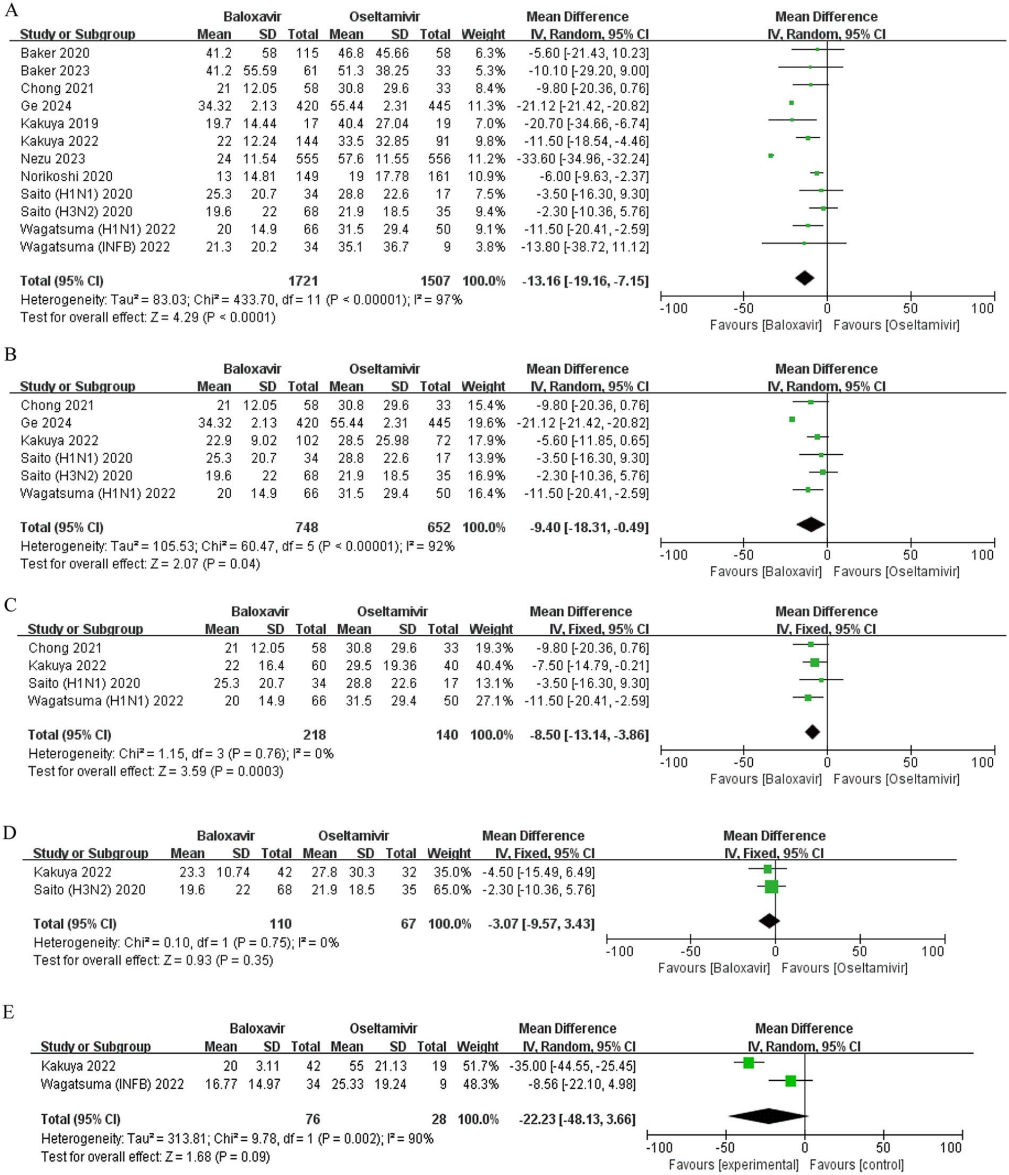
Forest plot of the effect of baloxavir marboxil vs. oseltamivir on the duration of fever in influenza patients. **(A)** Overall comparison. **(B)** Influenza A subgroup analysis. **(C)** Influenza A (H1N1) subgroup analysis. **(D)** Influenza A (H3N2) subgroup analysis. **(E)** Influenza B subgroup analysis.

Ten independent trials investigated fever duration disparities between baloxavir and oseltamivir (14–17, 19, 20, 22–25). Due to inconsistent conclusions, a random-effects model meta-analysis was employed to address substantial heterogeneity (*I*^2^ = 97%, *P* < 0.0001). Pooled results indicate that there is a significant difference in fever resolution between the two interventions (MD = −13.16 h, 95% CI: −19.16 to −7.15, *P* < 0.0001) (Figure 3A).

Subgroup analyses stratified by viral subtypes revealed divergent outcomes. For influenza A, there was significant inter group heterogeneity among the five studies (*I*^2^ = 92%, *P* < 0.0001), and quantitative synthesis of five studies showed baloxavir reduced fever duration by a mean difference of −9.40 h (95% CI: −18.31 to −0.49, *P* = 0.04) (Figure 3B) (14, 16, 17, 20, 25). Similarly, in H1N1 influenza cases, analysis of five trials demonstrated a significant reduction (MD = −8.50 h, 95% CI: −13.14 to −3.86, *P* = 0.0003) (Figure 3C) (14, 16, 20, 25). Conversely, no advantage was observed for H3N2 infections (MD = −3.07 h, 95% CI: −9.57 to 3.43, *P* = 0.35) (Figure 3D) (14, 20). Notably, baloxavir exhibited no advantage against influenza B, with an MD of −22.23 h (95% CI: −48.13 to 3.66, *P* = 0.09) (Figure 3E) (20, 25). Furthermore, a pediatric cohort study focusing on children aged 0–6 years confirmed accelerated fever resolution with baloxavir (*P* < 0.001), underscoring its potential clinical utility in younger populations (22).

### 3.4 Sensitivity analysis

In the comparison of fever duration differences, significant heterogeneity was observed between the baloxavir and oseltamivir groups. To investigate whether heterogeneity among studies was driven by individual trials, a sensitivity analysis was performed using sequential exclusion of each study. The results demonstrated no substantial heterogeneity and no directional changes in the pooled estimates, indicating that no single study was the primary source of heterogeneity and supporting the robustness of the findings. Detailed results are presented in Table 3.

**Table 3:** Pooled effect of fever duration outcomes between baloxavir and oseltamivir groups after sequential exclusion of individual studies.

First author	MD	95%CI	*P*	*I*^2^ (%)
Baker et al. (14)	−13.67	−19.87,−7.47	<0.00001	98
Baker et al. (15)	−13.33	−19.51,−7.15	<0.00001	98
Chong et al. (16)	−13.46	−19.75,−7.18	<0.00001	98
Ge et al. (17)	−11.79	−22.79,−0.78	<0.00001	97
Kakuya et al. (19)	−12.58	−18.83,−6.33	<0.00001	98
Kakuya et al. (20)	−13.33	−19.69,−6.97	<0.00001	98
Nezu et al. (22)	−10.56	−17.14,−3.97	<0.00001	91
Norikoshi et al. (23)	−14.09	−20.29,−7.88	<0.00001	97
Saito et al. (24)	−15.28	−21.77,−8.78	<0.00001	98
Wagatsuma et al. (25)	−13.29	−19.76,−6.82	<0.00001	98

### 3.5 Duration of symptoms

**Figure 4:**
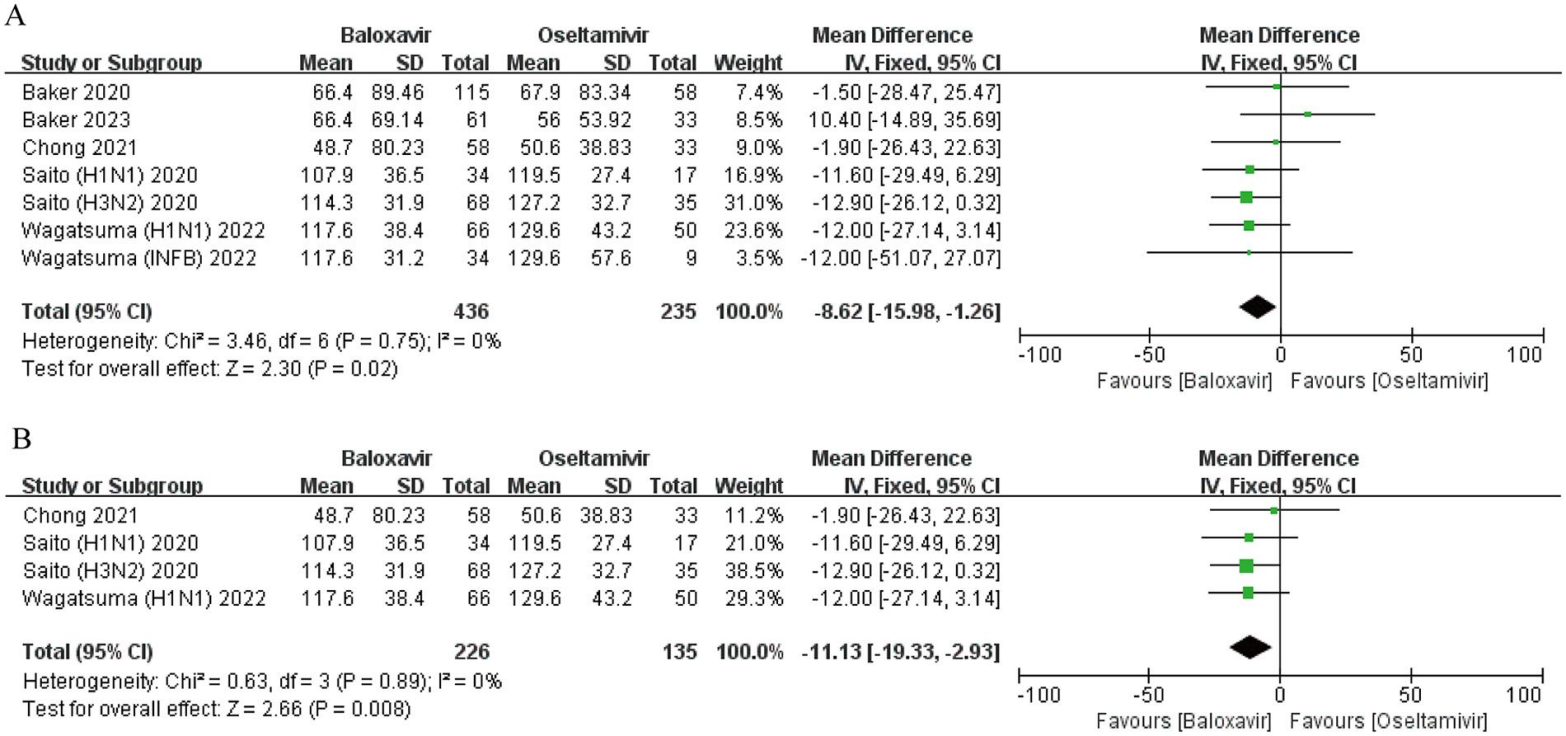
Forest plot of the effect of baloxavir marboxil vs. oseltamivir on the duration of symptoms in influenza patients. **(A)** Overall comparison. **(B)** Influenza A subgroup analysis.

In the assessment of clinical symptom duration across five prospective cohort investigations and randomized controlled trials (14–16, 24, 25), researchers conducted comparative analyses of therapeutic timelines between baloxavir marboxil and oseltamivir. The synthesized evidence from pooled statistical modeling demonstrated statistically significant advantages in symptom resolution timelines favoring the baloxavir cohort (mean difference = −8.62 h, 95% confidence interval: −15.98 to −1.26, *P* = 0.02), as visually represented in Figure 4A. Subsequent stratified examination revealed distinct therapeutic patterns across viral subtypes. For influenza A infections, meta-analytic synthesis of three clinical trials confirmed accelerated symptom alleviation in the baloxavir treatment arm (MD = −11.13 h, 95% CI: −19.34 to −2.93, *P* = 0.008), with detailed comparative metrics illustrated in Figure 4B (14, 16, 24, 25). In the influenza B subpopulation analysis, longitudinal observational data from a multicenter cohort study documented clinically meaningful reductions in median symptom persistence (4.9 ± 1.3 treatment days vs. 5.4 ± 2.4 control days, *P* < 0.01) (25), suggesting potential virological mechanism variations influencing therapeutic response durations.

### 3.6 Virological changes

Two RCT studies reported the changes in viral titers 24 h after treatment relative to baseline (14, 18). Both studies indicated that, compared to oseltamivir, baloxavir marboxil resulted in a greater reduction in influenza virus titers at 24 h from baseline. Specifically, Baker et al. found that the decrease in influenza virus titer in the baloxavir marboxil group was significantly greater than in the oseltamivir group (−3.59 ± 1.34) log_10_ tissue culture infective dose (TCID_50_)/ml vs. (−1.79 ± 1.54) log10 TCID_50_/ml (14). Ison et al.'s study showed that the viral titer in the baloxavir marboxil group decreased by an average of 3.36 log_10_ TCID_50_/ml from baseline, compared to a 1.76 log_10_ TCID_50_/ml reduction in the oseltamivir group (18).

A high-quality cohort study reported the viral clearance rate (21). In the influenza A subgroup, the median viral clearance rate in the baloxavir marboxil group was significantly higher than in the oseltamivir group [0.81 (0.52, 1.12) log_10_ copies/day vs. 0.63 (0.44, 0.93) log_10_ copies/day, *P* = 0.007]. In the influenza B subgroup, there were no significant differences in viral clearance rates between the baloxavir marboxil group [0.77 (0.38, 1.04) log10 copies/day] and the oseltamivir group [0.64 (0.48, 0.86) log10 copies/day](*P* > 0.05). Since the reduction in viral titer (log₁₀ TCID₅₀/mL) and viral clearance rate (log₁₀ copies/day) represent distinct biological constructs with incompatible units, the virological endpoints were deemed unsuitable for meta-analysis in the present study.

### 3.7 Viral drug resistance mutations

Three studies reported resistance mutations to baloxavir marboxil (14, 24, 25). Baker et al. (14) observed that among 57 children with sequenced baseline and post-treatment samples, 9 were infected with influenza A (H3N2) and 2 with H1N1pdm09. The incidence of PA/I38X substitutions was higher in children aged 1–<5 years [5/16 (31.3%)] compared to those aged 5–<12 years [6/41 (14.6%)]. Saito et al. (24) reported a median fever duration of 20 h (range: 0–96 h) in 13 cases with PA variants; the median duration of all symptoms was 121 h (range: 26–147 h), with no significant differences in fever or symptom duration between patients with and without PA/I38X mutant viruses. Wagatsuma et al. (25) reported frequencies of 4.5% (1/22) in the A(H1N1)pdm09 group and 0.0% (0/12) in the B/Victoria lineage group. Due to the limited data on viral resistance mutations and the incompatible units of the reported subgroup data, a meta-analysis of viral resistance mutation endpoints was not performed.

### 3.8 Safety evaluation

#### 3.8.1 Adverse events (AEs) incidence

**Figure 5:**
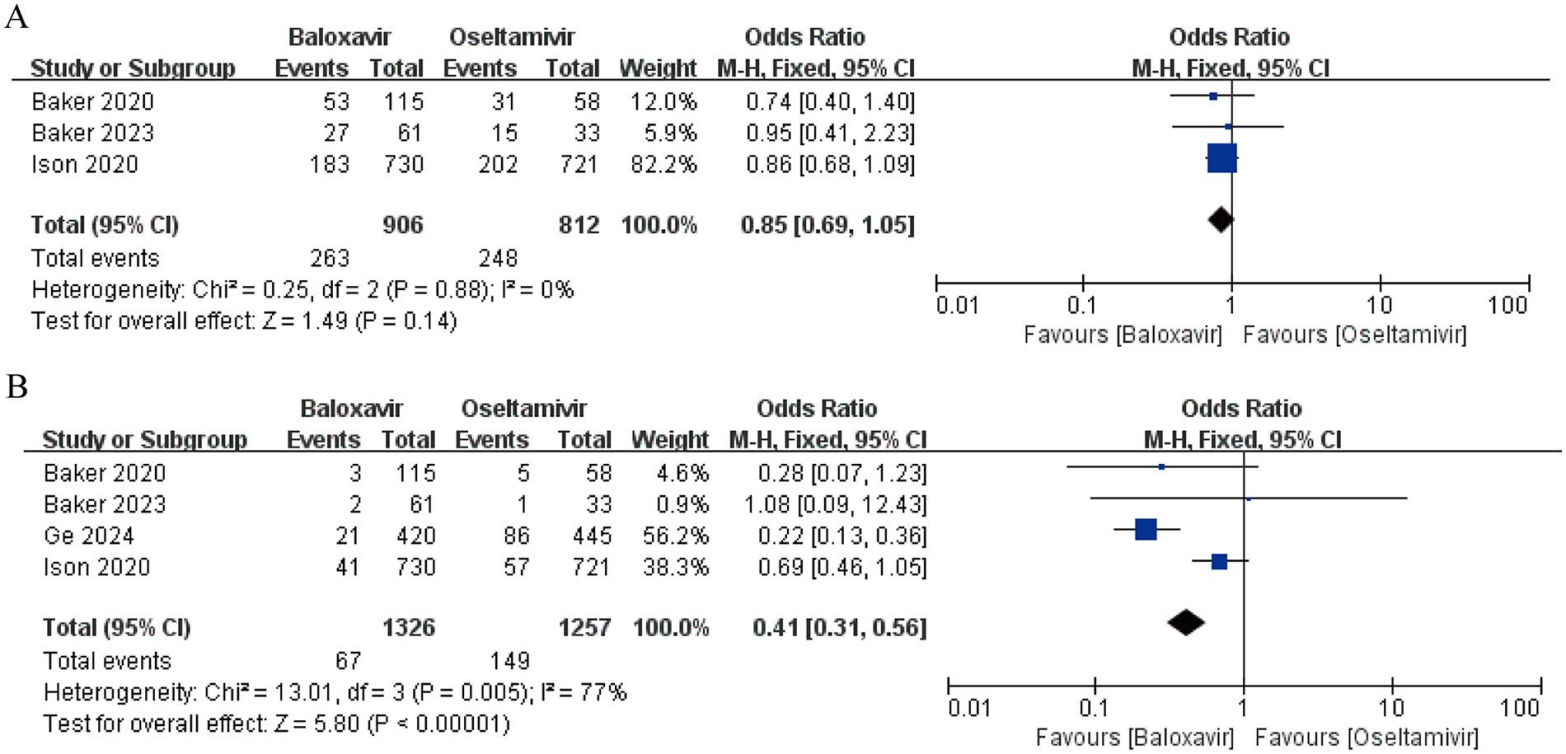
Forest plot of the incidence of adverse events with baloxavir marboxil vs. oseltamivir. **(A)** overall incidence of adverse events. **(B)** Incidence of drug-related adverse events.

Three medium- to high-quality RCT studies reported the incidence of AEs (14, 15, 18). Baker et al. demonstrated that the occurrence of AEs in the baloxavir group (46.1%) was lower than in the oseltamivir cohort (53.4%) (14). In contrast, Ison et al. observed AE rates of 25% and 28% for baloxavir and oseltamivir, respectively (18), while another study by Baker et al. reported no statistically significant intergroup disparity, with both therapies showing a 44% AE prevalence (15). A pooled analysis of these datasets indicated no significant difference in AE incidence between the two agents (OR = 0.85, 95% CI: 0.69–1.05, *P* = 0.14) (Figure 5A). Notably, no fatalities were documented across studies. The AE spectrum diverged between treatments: baloxavir was predominantly associated with cutaneous reactions (rash), gastrointestinal disturbances (diarrhea), and sinus inflammation, whereas oseltamivir exhibited higher frequencies of emesis, nausea, and middle ear infections.

#### 3.8.2 Drug-related adverse events (DRAEs) incidence

Four studies reported the incidence of DRAEs (14, 15, 17, 18). Baker et al. identified a lower DRAE incidence with baloxavir (2.6% vs. 8.6% for oseltamivir), while Ison et al. noted rates of 6.0% and 3.0% in the respective treatment arms. A subsequent investigation by Baker et al. revealed equivalent DRAE rates (3%) for both therapies. There was substantial heterogeneity among the studies (*I*^2^ = 77%). Meta-analysis demonstrated a clinically meaningful reduction in DRAE risk with baloxavir (OR = 0.41, 95% CI: 0.31–0.56, *P* < 0.0001) (Figure 5B). Severe DRAEs also favored baloxavir, with incidence rates of 0.68% vs. 1.11% for oseltamivir. These findings suggest baloxavir's tolerability profile regarding treatment-emergent complications.

## 4 Discussion

This article included 12 studies on the efficacy and safety of baloxavir marboxil in treating pediatric influenza, providing an in-depth analysis of its application in children with different influenza subtypes. Regarding efficacy, compared to NAIs, baloxavir marboxil demonstrated distinct strain-specific antiviral effects. For influenza A, its overall effectiveness was influenced by inter-subtype variations and high heterogeneity. Specifically, although the pooled analysis of influenza A indicated that baloxavir shortened the duration of fever, the extremely high heterogeneity (*I*^2^ = 92%) suggested the presence of important confounding factors. Subsequent subgroup analysis successfully deconstructed this heterogeneity and clarified the efficacy differences: baloxavir provided clear benefits for H1N1 infections, significantly reducing the duration of fever; however, no similar advantage was observed in H3N2 infections. This strain-specific variation in efficacy may be related to differences in viral replication characteristics, drug susceptibility, or host immune responses among influenza subtypes. Therefore, based on the strain-specific results, baloxavir marboxil can be considered significantly effective against influenza A (H1N1) in children, with its broad-spectrum antiviral activity primarily manifested in the effective suppression of these specific virus subtypes.

In terms of virological changes, limited existing studies suggest that baloxavir marboxil may have advantages in reducing influenza viral load, but caution should be exercised when interpreting it. However, it must be specifically noted that this study did not perform a quantitative pooled analysis of virological endpoints. This is primarily due to fundamental differences in the virological metrics used across studies—two randomized controlled trials reported viral titer changes in log₁₀ TCID₅₀/mL, while a cohort study measured viral clearance rates in log₁₀ copies/day. These metrics reflect different biological characteristics of the virus and are dimensionally incompatible, preventing effective meta-analytic pooling of the data. Despite this measurement heterogeneity, the independent results from the various studies demonstrate directional consistency: baloxavir marboxil was more effective than oseltamivir in reducing the influenza virus titer 24 h post-treatment and in shortening the duration of viral shedding. Baloxavir also exhibited a higher median virus clearance rate in children with influenza A (26). However, given the limited sources and methodological variations in virological data, these findings should be regarded as preliminary evidence. While the rapid reduction in viral load is theoretically appealing, its precise correlation with clinical symptom improvement requires further confirmation through additional studies. The mechanism behind these results may be related to the drug's mode of action. Oseltamivir works by inhibiting the neuraminidase on the surface of the influenza virus, preventing the release of viral particles from cells, thereby inhibiting virus transmission (10). In contrast, baloxavir marboxil inhibits the cap-dependent endonuclease required for viral replication, which more effectively blocks the virus lifecycle at an earlier stage.

It is particularly important to note that although baloxavir marboxil demonstrates good efficacy in children, the issue of drug resistance warrants serious attention. Some of the studies included in this analysis (14, 24, 25) reported the occurrence of PA/I38T mutations, which is consistent with existing evidence—the incidence of baloxavir marboxil-related resistance mutations (primarily PA/I38T/M/F and E23K) in pediatric patients (9%–23%) is significantly higher than in adults. These resistance mutations have a clear impact on clinical outcomes: multiple studies have shown that viruses carrying PA/I38T and other mutations are associated with viral load rebound, prolonged duration of fever and respiratory symptoms, and may lead to clinical treatment failure. Furthermore, experimental studies have confirmed that variants carrying PA/I38T retain their transmission capability, posing a potential public health risk. Therefore, resistance monitoring should be a core consideration in the clinical use of baloxavir marboxil in pediatric patients.

Regarding safety, baloxavir marboxil is generally well tolerated. The pooled analysis of this study revealed that although there was no significant difference in the overall incidence of adverse events (AEs) between baloxavir marboxil and oseltamivir (OR = 0.85, 95% CI: 0.69–1.05, *P* = 0.14), baloxavir marboxil demonstrated a significant advantage in terms of drug-related adverse events (DRAEs) (OR = 0.41, 95% CI: 0.31–0.56, *P* < 0.0001). Analysis of the specific adverse event profiles indicated that baloxavir marboxil was primarily associated with skin reactions (rash), gastrointestinal disorders (diarrhea), and sinus inflammation, while oseltamivir was linked to a higher frequency of vomiting, nausea, and middle ear infections. It should be noted that the U.S. FDA labeling for baloxavir warns of potential risks such as allergies, urticaria, angioedema, and erythema multiforme (12, 27). Although these serious skin reactions are rare in clinical studies, vigilance and enhanced monitoring in real-world applications remain necessary. In contrast, oseltamivir's common AEs include nausea, vomiting, and otitis media. The FDA also warns that oseltamivir may cause severe allergic skin reactions and neuropsychiatric events, particularly in pediatric patients. Based on the significantly lower risk of DRAEs observed in this study (incidence of severe DRAEs: 0.68% for baloxavir marboxil vs. 1.11% for oseltamivir), coupled with the immature physiological functions and metabolic capacity in children, baloxavir marboxil may indeed represent a preferable therapeutic option for pediatric influenza treatment. However, several critical real-world issues must be acknowledged: first, safety data for children under 5 years old, particularly those under 2 years, remain very limited; second, the issue of prolonged viral shedding associated with drug resistance warrants attention, as viruses carrying PA/I38T and other mutations may lead to extended viral clearance time and increased transmission risk; furthermore, although this study did not conduct a pharmacoeconomic evaluation, the cost-effectiveness comparison between baloxavir and oseltamivir is an important consideration in healthcare decision-making, and more real-world studies are needed to assess its long-term economic value. Consequently, long-term safety data for baloxavir in children is still insufficient, and close monitoring for adverse effects is necessary during use.

This study has several limitations. First, there were inconsistencies in outcome definitions (such as criteria for symptom resolution) and laboratory testing methods across the studies, making some data difficult to compare directly. It is particularly noteworthy that 8 out of the 12 studies included in this analysis were conducted in Japan. This geographic clustering may limit the generalizability of our findings to other regions and populations, as there may be variations in viral subtype distribution, medical practices, and host genetic backgrounds across different geographic areas. More importantly, the literature included in this study provides insufficient reporting on specific important subgroups, particularly infants and young children under 5 years of age, and lacks long-term follow-up data based on real-world settings. This limitation restricts our ability to evaluate the long-term safety and efficacy of baloxavir in these key populations. Furthermore, as the original studies did not provide stratified data based on disease severity (such as mild vs. moderate cases), underlying patient risk status (e.g., high-risk vs. otherwise healthy children), or healthcare settings (e.g., outpatient vs. inpatient), we were unable to perform corresponding subgroup analyses to evaluate the efficacy differences of baloxavir across these key clinical categories. This limitation somewhat diminishes the value of our findings in guiding precision clinical decision-making. Additionally, due to significant variations in the reporting of virological endpoints, we were unable to effectively perform quantitative synthesis of virological outcomes such as viral titer and viral clearance rate. On the analytical level, the substantial differences in study types among the included trials led to high heterogeneity in several meta-analyses (for instance, I² values exceeding 90% for primary efficacy endpoints), which may affect the robustness of the pooled results. Simultaneously, there is a lack of high-quality randomized controlled trials (RCTs), with only four of the 12 included studies being RCTs-along with their generally insufficient sample sizes, has constrained the statistical power of the analyses. Finally, due to the relatively recent approval of baloxavir marboxil, comprehensive data on its potential for drug resistance and long-term adverse effects in pediatric populations remain limited. Therefore, further well-designed clinical trials with long-term follow-up are needed to strengthen the evidence base.

In conclusion, baloxavir marboxil shows promising efficacy and safety in the treatment of pediatric influenza and provides a new therapeutic option for clinical practice. Based on the findings of this study, combined with the IDSA/ESPID guidelines recommending early antiviral treatment, baloxavir is particularly suitable for children with influenza A (H1N1) and influenza B, providing clinicians with an alternative option beyond neuraminidase inhibitors. However, due to the limitations of the included studies, and considering the CDC and WHO requirements for long-term safety data on new antiviral medications, continued monitoring of the clinical efficacy and adverse effects of baloxavir marboxil in large-scale, prospective real-world studies in pediatric patients to obtain more comprehensive and reliable evidence for its use.

## Data Availability

The raw data supporting the conclusions of this article will be made available by the authors, without undue reservation.
